# CHD@ZJU: a knowledgebase providing network-based research platform on coronary heart disease

**DOI:** 10.1093/database/bat047

**Published:** 2013-07-01

**Authors:** Leihong Wu, Xiang Li, Jihong Yang, Yufeng Liu, Xiaohui Fan, Yiyu Cheng

**Affiliations:** Pharmaceutical Informatics Institute, College of Pharmaceutical Sciences, Zhejiang University, Hangzhou 310058, China

## Abstract

Coronary heart disease (CHD), the leading cause of global morbidity and mortality in adults, has been reported to be associated with hundreds of genes. A comprehensive understanding of the CHD-related genes and their corresponding interactions is essential to advance the translational research on CHD. Accordingly, we construct this knowledgebase, CHD@ZJU, which records CHD-related information (genes, pathways, drugs and references) collected from different resources and through text-mining method followed by manual confirmation. In current release, CHD@ZJU contains 660 CHD-related genes, 45 common pathways and 1405 drugs accompanied with >8000 supporting references. Almost half of the genes collected in CHD@ZJU were novel to other publicly available CHD databases. Additionally, CHD@ZJU incorporated the protein–protein interactions to investigate the cross-talk within the pathways from a multi-layer network view. These functions offered by CHD@ZJU would allow researchers to dissect the molecular mechanism of CHD in a systematic manner and therefore facilitate the research on CHD-related multi-target therapeutic discovery.

**Database URL:** http://tcm.zju.edu.cn/chd/

## Introduction

Coronary heart disease (CHD), also known as coronary artery disease (CAD), is the most common type of heart disease and the leading cause of global morbidity and mortality in adults (1–3). As a complex disease, CHD has been reported to be associated with hundreds of genes. Therefore, a comprehensive understanding of the CHD-related genes and their corresponding interactions is essential to advance the translational research on CHD.

So far, there are several databases that have collected gene information related to CHD. For instance, Rat Genome Database (RGD) (4), a knowledgebase primarily designed for rat genomic research, has provided genetic information associated with cardiovascular disease with literature proofs. In its current release of RGD (up to 2013/1/23), 1078 genes have been linked to cardiovascular disease. Another database is CADgene (5), which collected ∼300 CHD-related genes from literature. However, RGD covers the scope of cardiovascular disease that is broader than our particular interests on CHD, and the related number of gene would be reduced to 166 if we choose only coronary disease sub-category. On the other hand, CADgene focuses more on single nucleotide polymorphism of CHD genes. More importantly, these databases solely focus on CHD-related genes, such as sequences, chromosome locations or functions, without considering the critical genes–genes relationships that clearly play an important role in their cellular activities and pathological progression of CHD. As a matter of fact, with the rapid development of system biology and network pharmacology, interactome and diseasome have become hot research areas to systematically reflect the network relationships in biological system (6). A better understanding of diseasome network, such as protein–protein interaction network, might lead to the identification of disease biomarkers, elucidation of underlying mechanisms and new drug discovery. However, to our knowledge, there is no study to date reporting the diseasome network of CHD.

Here, we developed CHD@ZJU, a curated knowledgebase to provide a network-based research platform for CHD. Through text-mining technique followed by manual confirmation, we extracted 660 CHD-related genes from ∼90 000 abstracts of CHD-related articles. CHD@ZJU also mapped these CHD-related genes into 45 common pathways, including signal transduction, cellular process, organismal systems and human disease pathways from KEGG database. CHD@ZJU was specially designed for CHD diseasome study by integrating human protein-protein interaction (PPI) information from HPRD (release 9) (7) and BioGRID (version 3.2) (8) database. Additionally, drugs related to CHD-related genes were also imported from DrugBank. With CHD@ZJU, we hope to provide an integrated platform to facilitate the research on CHD, especially for molecular mechanism dissection and novel therapeutics discovery.

## Database Construction

**Figure 1.**
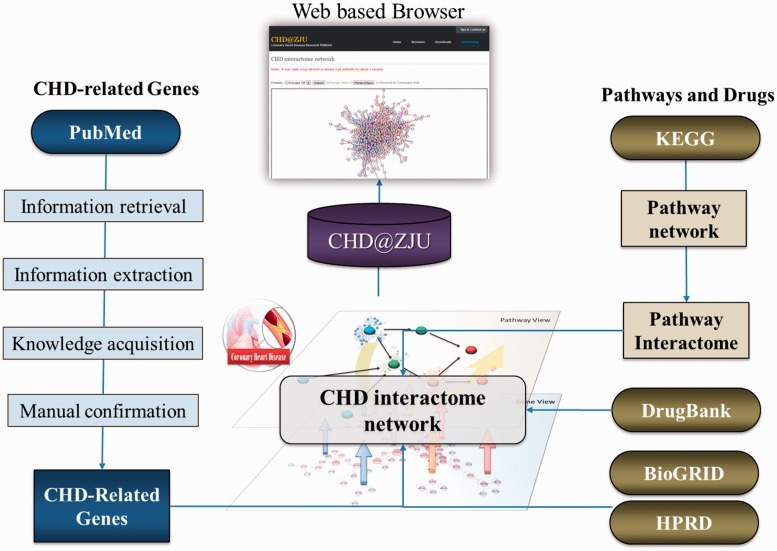
Procedure for CHD@ZJU construction. CHD-related genes were extracted with text-mining technique and manual confirmation. PPI, pathway and drugs information were then collected from public resources such as KEGG and HPRD. Interactome network of every pathway was constructed based on their corresponding genes and related PPIs, and the whole CHD diseasome network was then constructed with all CHD-related genes. With CHD@ZJU, users could find information related to CHD from gene, pathway and the whole biological network level.

The procedure of CHD@ZJU construction is shown in Figure 1. Briefly, we extracted CHD-related genes with text-mining technique and manual confirmation and collected PPI, Pathway and drugs information from public resources. Interactome network of every pathway and the whole CHD diseasome network were then constructed. CHD@ZJU also provides online interface to compare or merge interactome network with multiple pathways and to construct user-defined diseasome network with a set of specified CHD-related genes.

### CHD-related genes curation

The overall workflow to collect CHD-related genes could be generally divided into four steps: (i) information retrieval, (ii) information extraction, (iii) knowledge acquisition and (iv) manually confirmation.

In information retrieval section, CHD-related articles were retrieved from PubMed based on the keywords ‘CHD’, with the restriction on publish date being from 1 January 2000 to 23 January 2013. As a result, 115 898 articles were found, and their abstracts were downloaded for text mining. After removing the articles without abstracts, only 88 396 abstracts remained for further study.

Keywords potentially linked with genes were extracted from these abstracts in information extraction section. After ignoring those keywords starting with numbers, 101 402 keywords were resulted from this step. ArrayTrack was then used to identify related genes of these keywords from the gene library and remove the redundancy. In all, 4674 genes were then found by this knowledge acquisition procedure.

To validate the accuracy of this information, these 4674 genes were put back into context of original CHD-related articles, and only genes with official names and specific descriptions were kept, which reduced the number of related genes to 1247. The accurate relevance between these 1247 genes and CHD was then confirmed by manually reading their related abstracts and even the whole article. Some genes would be filtered as they represent other meanings, such as gene CAD (it means carbamoyl-phosphate synthetase 2), with the same abbreviation of coronary arterial disease. In this section, three reviewers independently examined the genes, and only consensus genes from all reviewers were defined as CHD-related genes in the final knowledgebase. At the end of this section, 660 CHD-related genes were validated by all three reviewers, with at least one supporting reference.

By our approach, all CHD-related genes were then compared with 1078 known cardiovascular genes in RGD cardiovascular diseases database. In all, 370 overlapped genes were found, and there were 290 genes that were not included in RGD database, but were supported by literature. For instance, polymorphism of a novel gene, ABCG1, was reported to be associated with the susceptibility and severity of coronary atherosclerotic disease (9). Previous study also indicated that ABCG1 played an important role in high-density lipoprotein metabolism (10), which suggests a strong correlation of this gene with cardiovascular disease (11). Therefore, according to our database and reference validation, ABCG1 would have high potential to be a new gene associated with CHD study. During the preparation of this work, a new version of RGD database was released, and another 17 CHD-related genes now were supported by RGD database, which demonstrated our algorithm to find CHD-related genes was promising.

### Pathway and drug information curation

Forty-five common human pathways including signal transduction, cellular processes, immune system, endocrine system, circulatory system and cardiovascular diseases were collected and downloaded from Kyoto Encyclopedia of Genes and Genomes (KEGG) database. Based on our previous studies related to CHD, these related pathways were particularly selected and included in our CHD@ZJU database for the first release. More pathways would also be incorporated for future updates.

Genes involved in these pathways were also collected, and then BioGRID and Human Protein Reference Database (HPRD) database were used to reveal the possible gene interactions. As a result, 27 554 and 9693 PPI interactions from BioGRID and HPRD were imported into CHD@ZJU. The genes in pathways would be considered as CHD-related if they were present in our list of CHD-related genes. These labels would be useful to represent the locations of CHD-related genes in the pathway interactome network.

A total of 1405 drugs involved with CHD-related genes were retrieved from DrugBank in CHD@ZJU, which included various types of biological and small molecule drugs, such as approved, nutraceutical, experimental, illicit and withdrawn drugs.

### Query interface

**Figure 2.**
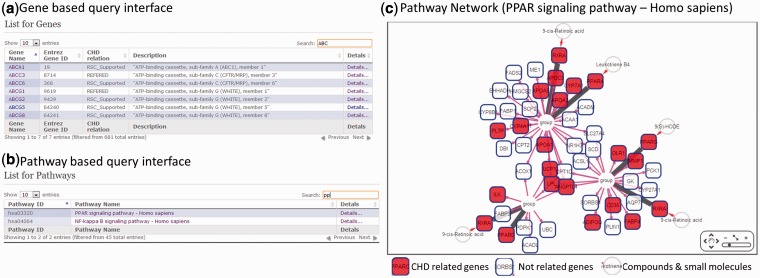
Query interface in CHD@ZJU. (**A**) Gene-based query interface; (**B**) pathway-based query interface; (**C**) network interactions of PPAR signaling pathway (homo sapiens). Interaction data are from KEGG database.

CHD@ZJU offered two query interfaces for users to explore CHD-related genes and corresponding information from our integrated database. The gene-based query interface was designed for users whose research focused on the CHD-related genes directly. We offered an interactive query table plug-in to search gene with its gene name, Entrez ID or detailed description (Figure 2a). For instance, if users were interested in gene ABCA1, an input of ‘ABCA1’, ‘19’ or ‘ATP-binding cassette’ could all be used to narrow down the table contents and eventually remain ABCA1 gene in the table. Under the entry of ABCA1, CHD@ZJU provided the supporting references and highlighted the corresponding sentence to relate ABCA1 with CHD. Three additional information about the relationship between ABCA1 and CHD was also provided in the information page of ABCA1. First, a complete of reference list related with ABCA1 was generated by text-mining approaches in all CHD articles. Some unavoidable mistakes might exist in these references retrieved by automated technology; however, it still provides useful information for users who want to examine the relations between ABCA1 and CHD in detail. The information about PPI interactions with ABCA1 was displayed with related references and interaction types from HPRD and BioGRID Databases. In addition, the information about ABCA1-related drugs would also be displayed if available from DrugBank.

The second query interface was pathway-based interface especially for users who were interested in CHD-related pathways and their relationships. Multiple pathways have been suggested to contribute to complex mechanisms of CHD onsets and progressions. Therefore, we provided an interface for users to browse for a specific pathway (Figure 2b) and isolate the location of CHD-related genes in this pathway and interactome PPI network. In general, the pathway information and connections between pathway elements were downloaded and imported from KEGG Database. CHD-related genes would be highlighted in the pathway map (Figure 2c).

## Database Description and Utility

### Pathway interactome

Pathway interactome is different from pathway network, as the pathway network mostly contains only experimentally validated information and could involve small molecules or ions, such as Ca^2+^. Although this kind of information is important to understand current pathways and diseases, information about relationship between genes involved in the pathways was deficiently present. With knowledge about PPI interactions between pathway-related genes, interactome would offer novel perspective to study the potential cross-talk among the pathways. Previous study suggests integrated information from different PPI databases would produce better and more robust PPI networks; therefore, we constructed all interactome for 45 human pathways with both BioGRID and HPRD PPI interaction information. In addition, a sub-pathway could be extracted with CHD-related genes for close inspections.

**Figure 3.**
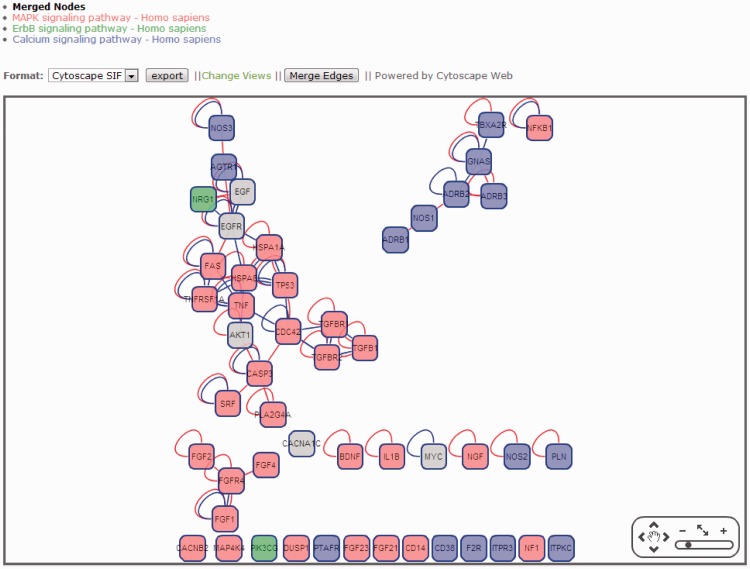
A merged pathway interactome network with MAPK, ErbB and calcium signaling pathways in Homo sapiens. Red nodes were genes involved in MAPK signaling pathways, blue nodes were genes involved in calcium signaling pathway and green nodes were genes involved in ErbB signaling pathway. Gray nodes were involved in multiple pathways.

In CHD@ZJU, we also provided a novel function for users to construct an integrated interactome network with multiple pathways, which might be useful to reveal the connections between multiple pathways and find their relationships with CHD disease (Figure 3). In the merged network, all genes were colored according to their belonged pathway, and genes involved in multiple pathways were marked as gray nodes. These overlapping nodes might play critical roles in pathway communication or cross-talk.

### CHD diseasome

**Figure 4.**
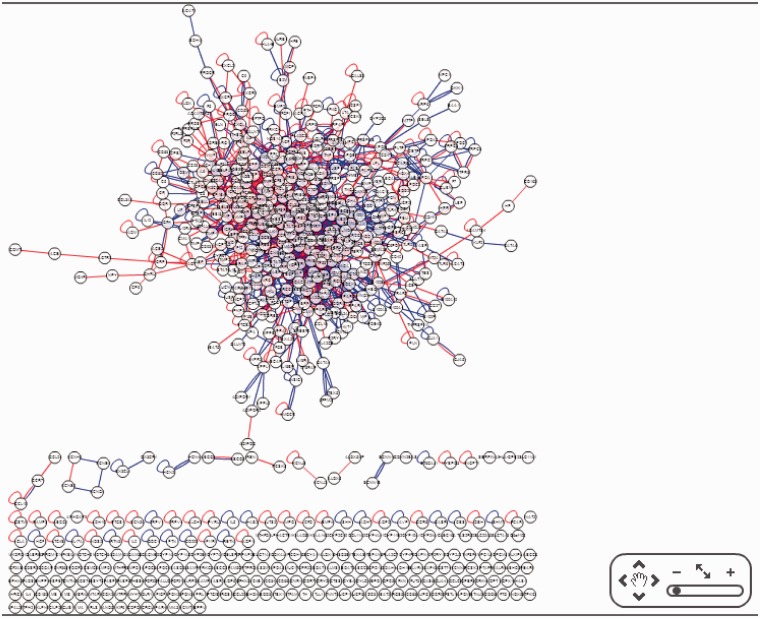
CHD diseasome in the human interactome. The giant module contained 428 CHD-related genes with 1495 connections.

CHD@ZJU also provided users to view the whole CHD interactome network that included all 660 CHD-related genes (Figure 4), or the customized interactome network with selected genes. The function of customized interactome network was mainly designed for users interested in the relationships between a small group of CHD-related genes. The CHD interactome network could also be used as CHD disease module (CHD diseasome), as it was the sub-network of the whole-human interactome.

We were fully aware that this CHD diseasome was still far from completion in the current version, as there were a large number of discrete nodes disconnected with the giant component of interactome. The biggest sub-network of CHD diseasome contained 428 genes and 1495 connections, which meant there were still 36.7% (242 CHD-related genes) not connected with this sub-network. One possible explanation is that multiple independent pathways and mechanisms for CHD might not interact in the biological system. Our opinion on this observation would be incomplete information collected about CHD and insufficient understanding about pathological progress and molecular mechanisms for this disease. Therefore, construction of this CHD diseasome would be a continuous process with integrating new information about CHD databases and most recent scientific discovery about CHD.

Although the CHD diseasome was not fully completed, we can discover some useful information by network analysis. We defined the interactome as an undirected network, and topological attributes for all genes in the giant component were then measured by Cytoscape (12). Table 1 listed the top and bottom five nodes in term of their betweenness scores. In general, genes at the top of the list were strongly correlated with CHD and showed multiple functions in biological system. For instance, polymorphism in EGFR gene was associated with the risk of acute coronary syndrome (13), and its corresponding protein epidermal growth factor receptor (EGFR) has been reported to be associated with the growth of angiotensin II-induced vascular smooth muscle cells (14). EGFR also was proved to be related with vasospastic response to endothelin (15). These results indicated the important role EGFR may play in CHD-related mechanisms. On the other side, nodes with lower betweenness and degree might be considered as pheripheral indicators of CHD. In our CHD diseasome, COMT gene was located as a vertex of a long chain, which only interacted with angiotension converting enzyme. Previous studies show Catechol-O-methyl transferase (COMT) was associated with systematic atherosclerosis in elderly Japanese individuals (16) and could modify the effect of coffee intake on incidence of acute coronary events (17). Therefore, it was likely that COMT was a specific influence factor in CHD.

**Table 1.** Top nodes ranked by betweenness

Entrez ID	Gene_name	Betweenness	Degree
1956	EGFR	0.121 596	47
3312	HSPA8	0.117 194	36
7157	TP53	0.10 533	67
207	AKT1	0.095 045	39
6774	STAT3	0.08 803	41
813	CALU	0	1
977	CD151	0	1
9332	CD163	0	1
1281	COL3A1	0	1
1312	COMT	0	1

### Data access and download

All CHD-related genes and its supported references could be freely accessed and downloaded in CHD@ZJU; we also provided interface to download network with multiple formats, such as.sif,.png and so forth. In current version, registration in our website is not required for users to access all the functions.

## Conclusion

To gain better understanding of CHD from a systematic and network view, we have constructed CHD@ZJU, a curated knowledgebase to provide a network-based study platform. In the current version, we curated 660 CHD-related genes, 45 pathways and 1405 related drugs, along with >37 000 PPI interactions. As described earlier in the text, CHD-related genes were retrieved from literature and manually validated, and their locations in human interactome were provided. CHD@ZJU offered pathway functional modules and CHD diseasome in the human interactome for users to study CHD at network and molecular levels.

## Funding

National S&T Major Project (No. 2012ZX09503001), Fundamental Research Funds for the Central Universities and Program for New Century Excellent Talents in University (NCET-12-0488). Funding for open access charge: National S&T Major Project (No. 2012ZX09503001).

*Conflict of interest*. None declared.
